# Supplementary material for: Getting More Out of Biomedical Documents with GATE's Full Lifecycle Open Source Text Analytics
Source: PLoS Comput Biol. 2013 Feb 7;9(2):e1002854. doi: 10.1371/journal.pcbi.1002854 (PMC3567135; doi:10.1371/journal.pcbi.1002854)
Supplement: Dataset S1 — GATE software. Dataset S1 bundled with this paper contains a distribution of GATE (or see http://gate.ac.uk/download/). (TGZ) [file pcbi.1002854.s001.tgz › plos-gate/plugins/Gazetteer_LKB/doc/dictionary_lifecycle.html]

The Large KB gazetteer - Developer Documentation - Dictionary Lifecycle


---

Last Published: 2009-10-17

---

##### Large KB Gazetteer

- Overview
- Usage
- Q & A
- GATE Compatibility
- GATE Gazetteer Feature Comparison
- Semantic Enrichment PR
- Samples

##### Developer Documentation

- **Dictionary Lifecycle**

Multiple instances of the large KB gazetteer processing resource share the same dictionary, thus saving memory when parallel annotation is implemented. The dictionary lifecycle specification explains in details how and when dictionaries are loaded during the initialization of the PR. This text is aimed at advanced users, that seek to understand the details, and developers, that intends to extends the gazetteer.

This is a work in progress. Contact the team if you need immediate information on that topic.

---

©
2009
Ontotext AD

---
